# Supplementary material for: The Experience of Women Giving Birth after Cesarean Section—A Longitudinal Observational Study
Source: Healthcare (Basel). 2023 Jun 20;11(12):1806. doi: 10.3390/healthcare11121806 (PMC10297878; doi:10.3390/healthcare11121806)
Supplement: Supplementary file 1 [file healthcare-11-01806-s001.zip › appendixes word/Appendix A.docx]

**[Text]**

We would like to invite you to participate in a study conducted by St. Sophia Hospital in Warsaw in cooperation with the CMKP Department of Reproductive Health, which aims to examine the attitudes and motivation of women to the mode of delivery after previous caesarean section.

Childbirth after previous caesarean section is still a little known and emotional topic in our society. Therefore, we kindly ask you to fill out the following questionnaire about your previous deliveries (including previous caesarean sections) and your motivation and your hopes for your next delivery (natural childbirth or caesarean section).

The questionnaire is anonymous. The data obtained will be used for research purposes.

The data collected will be used for research purposes and the findings will help to improve the quality of care provided to pregnant women.

The questionnaire will take about 10 minutes to complete.

**S1.** **[O]**

**Current gestational week? Please enter a number.**

[H1. [Z, max. 1]*](#_Toc22906) **[How many cesarean sections have you had so far?](#_Toc22906)**

1. [1](#_Toc22906)
2. [2](#_Toc22907)
3. [3](#_Toc22908)
4. [4](#_Toc22909)
5. >4

**H2. [Z, max. 1]***

**Have you ever had a elective cesarean section (indication for the section was known before the start of labor)?**

1. Yes

### 2. No

*show H3, if H2=1*

**H3. [PO, max. 9]***

**What were the indications for elective cesarean section(s)? You may choose more than one answer.**

1. abnormal position of the fetus (e.g. pelvic, oblique)
2. suspicion of macrosomia, feto-pelvic disproportion
3. maternal diseases related to pregnancy (e.g. cholestasis, diabetes, hypertension)
4. cesarean scar dehiscence after a previous cesarean section
5. placental abnormalities (e.g., placenta previa, abnormal invasion)
6. non-obstetric indications (e.g. cardiological, psychiatric, ophthalmological)
7. fetal indications (e.g. heart defects contraindicated in vaginal birth)
8. the cesarean section was planned, but it was performed earlier (e.g. premature rupture of membranes, premature contractions)

**H4. [Z, max. 1]***

**Have you ever had an emergency cesarean section (indication for a section was diagnosed during labor)?**

1. Yes

## 2. No

*show H5, if H4=1*

**H5. [PO, max. 10]***

**What were the indications for emergency cesarean section(s)?**

1. abnormal position of the head, abnormal progress of the head into the birth canal (e.g.

asynclitism, face, brow, shoulder position)

1. lack of progress in the first stage of labor
2. lack of progress in the second stage of labor
3. Fetal distress, abnormal fetal heart rate (e.g. bradycardia)

5.abnormal test results (e.g. pre-eclampsia, cholestasis, etc.)

1. umbilical cord prolapse
2. uterine rupture
3. premature abruption of the placenta
4. Delivery disproportionate (e.g. too big baby, narrowed pelvis)

10.other?

**H6. [Z, max. 1]***

**Have you had skin-to-skin contact with your baby after a C-section(s)?**

- 1. Yes, always
  2. Yes, but not always

## 3. No

**H7. [Z, max. 1]***

**How would you assess your experience with lactation after cesarean section(s)?**

1. mostly very good
2. mostly good
3. mostly neither good nor bad
4. mostly bad
5. mostly very bad
6. different in each birth

**H8. [Z, max. 1]***

**Have you ever had a vaginal delivery ?**

1. Yes

### 2. No

*show H9, if H8=1*

**H9. [Z, max. 1]***

**Have you ever had a vaginal delivery after a cesarean section(s)?**

1. Yes

### 2. No

*show H10, jif H8=1*

**H10. [Z, max 1]***

**Have you had skin-to-skin contact with your baby after a vaginal delivery(s)?**

1. Yes, always
2. Yes, but not always
3. No

**H11. [Z, max. 1]***

**How would you assess your experience with lactation after vaginal delivery?**

### 1. mostly very good 2. mostly good 3. mostly neither good nor bad 4. mostly bad 5. mostly very bad

**K1. [Z, max. 1]***

**Please mark the one answer that best represents your opinion of childbirth from the following:** 1. A woman is able to give birth to a baby and usually no medical interventions are needed to do so

2. Childbirth is a normal physiological process, but medical interventions are necessary to manage the birth and provide the opportunity to deliver a healthy baby 3. Childbirth is always high risk for the baby and mother.

**W1. [Z, max. 1]***

**Would you agree with the following statements:**

|  | I strongly agree | I rather agree | I rather disagree | I strongly  disagree | I have no opinion |
| --- | --- | --- | --- | --- | --- |
| only a vaginal birth is a good birth |  |  |  |  |  |
| a woman is a better mother after a vaginal birth |  |  |  |  |  |
| a natural birth after a caesarean section is possible |  |  |  |  |  |
| a natural birth is too much effort and unnecessary confusion |  |  |  |  |  |
| postpartum recovery from a vaginal birth is easier compared to postpartum recovery from a caesarean section |  |  |  |  |  |
| caesarean section is easy and safe |  |  |  |  |  |
| caesarean section is safer for mother and baby compared to a vaginal birth |  |  |  |  |  |
| after a caesarean section, every subsequent birth must be a caesarean section |  |  |  |  |  |

**W2. [Z, max. 1]***

**Which mode of delivery do you prefer when you are currently pregnant?**

1. vaginal birth
2. caesarean section
3. I have to have a c-section for medical reasons
4. I have not yet thought about my preferred method of delivery in my current pregnancy

**W3. [Z, max. 1]***

**When did you decide on your preferred mode of birth?**

1. Before I was in the current pregnancy
2. As soon as I found out that I was pregnant
3. Before 13 weeks of current pregnancy
4. Between 14 and 27 weeks of current pregnancy
5. After 27 weeks of current pregnancy
6. I haven't made my decision yet

**W4. [Z, max. 6]***

**Did your obstetrician/midwife present:**

1. possibility of attempting a vaginal birth after caesarean section
2. possibility of not consenting to a vaginal birth after cesarean
3. the benefits of a vaginal birth after cesarean
4. the risks of attempting a vaginal birth by natural means after cesarean
5. advantages and disadvantages of cesarean section
6. My obstetrician/midwife did not present me with options. He/she made his/her own decision about the mode of delivery.

**W5. [Z, max. 1]***

**What attitudes about your preference for a mode of delivery in your current pregnancy do you take:?**

|  | enthuz  iasm | support | acceptance | indifference | disapproval | criticism | We did not talk |
| --- | --- | --- | --- | --- | --- | --- | --- |
| 1. Obstetrican |  |  |  |  |  |  |  |
| 2. Midwife |  |  |  |  |  |  |  |
| 3. Husband/partner |  |  |  |  |  |  |  |
| 4. Parents |  |  |  |  |  |  |  |
| 5. Family |  |  |  |  |  |  |  |
| 6. Friends |  |  |  |  |  |  |  |

**W6.** **[PO, max. 9]***

**Where did you get information about birth after the cesarean section? You may choose more than one answer.**

1. Physicians
2. Midwives
3. Scientific journals and books
4. Women's press, parenting guides
5. Internet - websites, blogs
6. Internet - social media (e.g., Facebook) 7. Family and friends
7. I wasn't looking for information on this
8. Other

**W7.** **[Z, max. 1]***

**What are you afraid of in the upcoming birth?**

|  | definitely yes | rather yes | rather not | definitely not | I have no opinion |
| --- | --- | --- | --- | --- | --- |
| 1. CS scar rupture |  |  |  |  |  |
| 2. Pain during vaginal birth |  |  |  |  |  |
| 3. Extensive perineal injuries |  |  |  |  |  |
| 4. Low Apgar Score of Newborn |  |  |  |  |  |
| 5. Postpartum hemorrhage |  |  |  |  |  |
| 6. Negative attitude of medical staff towards VBAC |  |  |  |  |  |
| 7. Disrespect my rights |  |  |  |  |  |
| 8. That the TOLAC will end in CS |  |  |  |  |  |
| 9. Hysterectomy |  |  |  |  |  |
| 10. Injury during CS |  |  |  |  |  |

**W8.** **[PO, max. 1]***

**What has had the greatest impact on your preference for the mode of delivery in your current pregnancy?**

1. the opportunities and risks associated with vaginal birth and repeat caesarean section
2. experience of previous vaginal births
3. experience of previous caesarean sections
4. opinion and indications of attending clinician
5. advice from other women having vaginal birth after cesarean section
6. opinions of family and relatives
7. other

**W9.** **[Z, max. 1]***

**What was your motivation in choosing the mode of birth?**

|  | definitely yes | rather yes | rather not | definitely not | I have no opinion |
| --- | --- | --- | --- | --- | --- |
| 1. Minimizing pain |  |  |  |  |  |
| 2. Convenience and predictability of ECS |  |  |  |  |  |
| 3. The extent of cesarean section |  |  |  |  |  |
| 4. A better bond between mother and child |  |  |  |  |  |
| 5. Ensuring better health for your child |  |  |  |  |  |
| 6. Ensuring better health of the mother |  |  |  |  |  |
| 7. Ensuring skin-to-skin contact |  |  |  |  |  |
| 8. Better conditions for breastfeeding |  |  |  |  |  |
| 9. Influence of the mode of childbirth on subsequent pregnancies and deliveries |  |  |  |  |  |
| 10. Faster recovery |  |  |  |  |  |
| 11. Less blood loss |  |  |  |  |  |
| 12. A sense of fulfillment. |  |  |  |  |  |
| 13. Strengthening the sense of femininity |  |  |  |  |  |
| 14. Improving your relationship with your partner |  |  |  |  |  |
| 15. Previous birth experiences |  |  |  |  |  |
| 16. The previous postpartum experience |  |  |  |  |  |

*show W10, if W3=1*

**W10.** **[Z, max. 1]***

**How do you feel about your decision to attempt a vaginal birth?**

1. I am sure it is the best decision
2. I want to try it - we'll see what happens
3. I still don't know if I made the right choice
4. I am seriously considering changing my decision

*show W11, if W3=2*

**W11.** **[Z, max. 1]***

**How do you feel about your decision not to consent to a vaginal birth?**

1. I am sure it is the best decision
2. I still don't know if I made the right choice
3. I am seriously considering changing my decision

**W12.** **[PO, max. 42]***

**How did you prepare for the birth after CS? You may choose more than one answer.**

1. Substantially - I read about it
2. I have prepared a birth plan
3. I read the stories of other births after CS
4. I signed up for support groups
5. I attended childbirth classes
6. I was physically active during my pregnancy
7. I attended yoga classes
8. I used psychotherapy
9. I used alternative medicine (e.g., acupressure, herbs)
10. I chose a physician to support my preferences
11. I chose a midwife to support my preferences
12. I chose a midwife to support my preferences
13. I have planned my birth in a hospital where the medical staff will respect my decision
14. Other

**M1.** **[Z, max. 1]***

**Age**

- 1. 18-20
  2. 21-25
  3. 26-30
  4. 31-35
  5. 36-40
  6. > 40

**M2.** **[Z, max. 1]***

**Education**

- 1. Primary
  2. Lower secondary
  3. Secondary

## 4. Higher

**M3.** **[Z, max. 1]***

**Residence**

1. A rural area
2. A small city (population less than 50,000)
3. A medium city (population between 50,000 – 99,999)
4. A large city (population between 100,000 – 499,999)
5. A very large city (population over 500,000)

**M4.** **[Z, max. 1]***

**Marital status**

1. Married
2. Partnership
3. Single
4. Divorced

**[Text]**

Thank you very much for completing the questionnaire and sharing your experiences, hopes, and concerns. The results of our analysis will be used to improve the quality of perinatal care for patients after cesarean section.
