# Supplementary material for: The Experience of Women Giving Birth after Cesarean Section—A Longitudinal Observational Study
Source: Healthcare (Basel). 2023 Jun 20;11(12):1806. doi: 10.3390/healthcare11121806 (PMC10297878; doi:10.3390/healthcare11121806)
Supplement: Supplementary file 1 [file healthcare-11-01806-s001.zip › appendixes word/APPENDIX B.docx]

The survey, part 2: ***Patients' motivations and attitudes about choosing a delivery route
after a previous cesarean section***

## LEGEND:

[O] Open

[PO] Semi-open

[Z] Closed

[Text] Text screen

[max 1] Maximum number of responses

* Mandatory question.

**[Text]**

Dear Sir or Madam,

## PART I Obstetrical History

**H1. [Z, max. 1]*.**

**Have you attempted natural childbirth after a cesarean section?**

1. No, I did not consent to a natural childbirth, I had a scheduled C-section
2. I wanted to attempt a natural childbirth, but it was not possible (no induction of labor, disqualification from childbirth in the last weeks of pregnancy). The pregnancy was completed by elective cesarean section.
3. Yes, I attempted, but it ended in an emergency cesarean section.
4. Yes, I attempted labor and gave birth by natural means.

*Display H2 if H1=2*

**H2. [PO, max. 7]*.**

**What was the main indication for an elective cesarean section?**

1. The labor did not start on its own. No possibility of labor induction
2. Unsuccessful induction of labor
3. Abnormal fetal position (e.g., pelvic, oblique)
4. Suspected large fetal weight, fetal-pelvic incommensurability
5. Thin scar from previous cesarean section
6. Non-obstetrical indications (e.g., cardiology, psychiatry, ophthalmology)
7. Indications from the fetus (e.g., heart defects preventing PSN)
8. I don't know
9. other, what?

*Display H3 if H1=3*

**H3. [PO, max. 10]*.**

**What were the indications for an emergency cesarean section?**

1. Abnormal positioning of the head, poor insertion of the head into the birth canal ( e.g. asynclitism, high straight standing of the head, facial positioning).
2. No progress of labor in the first period of labor
3. No progress of labor in the second period of labor
4. Threatening fetal asphyxia (e.g., drops in the baby's heart rate)
5. Abnormal test results (e.g., pre-eclampsia, cholestasis, etc.).
6. Umbilical cord prolapse
7. Threatening uterine rupture
8. Premature separation of the placenta
9. Unsuccessful induction of labor
10. I don't know
11. other, what?

**H4 [Z, max. 1]*.**

**How much did the baby weigh at birth?**

1. under 2000g
2. 2000g-2500g
3. 2501g-4000g
4. 4001-4500g
5. over 4500g

**H5 [Z, max. 1]*.**

**How many Apgar points was the baby rated at?**

1. 8-10 Apgar points
2. 4-7 Apgar points
3. 0-3 Apgar points

*Display H6 if H1=3 or 4*

**H6 [PO, max. 12]*.**

**Which methods of labor pain relief did you use? (You may mark more than one answer).**

1. Vertical positions
2. TENS
3. Massage
4. Aromatherapy
5. Music therapy
6. Breathing techniques
7. Water immersion (the birth itself did not take place in the water)
8. Use of the shower
9. Inhalation anesthesia (known as laughing gas)
10. Pharmacological anesthesia
11. Epidural anesthesia
12. None of the above
13. Other (what?..........)

*Display H7 if H1=3 or 4*

**H7 [Z, max. 1]*.**

**How was the baby's heart function monitored during delivery?**

1. Continuous KTG recording without mobility
2. Continuous KTG recording with mobility
3. I was offered continuous KTG recording but declined
4. Periodic auscultation of fetal heart rate and KTG once in a while

*Display H8 if H1=4*

**H8 [PO, max. 12]*.**

**Did you experience complications related to natural childbirth? If yes, what kind?**

1. There were no complications
2. Surgical delivery (forceps or obstetric vacuum)
3. Shoulder dystocia
4. Separation of the scar after cesarean section
5. Extensive perineal injuries (grade III and IV)
6. Increased bleeding from the genital tract (more than 500ml)
7. Blood transfusion
8. Removal of the uterus
9. Impaired wound healing after perineal suturing
10. Stopping puerperal feces
11. Uveitis
12. Other (what?................)

*Display H9 if H1=1 or 2 or 3*

**H9 [PO, max. 10]*.**

**Did you experience any complications related to the cesarean section? If so, which ones?**

1. I did not experience any complications
2. Separation of the scar after cesarean section
3. Increased blood loss (more than 1000ml)
4. Blood transfusion
5. Mechanical damage during surgery (e.g., ureter, bladder, bowel)
6. Removal of the uterus
7. Impaired wound healing after cesarean section
8. Stopping puerperal feces
9. Uveitis
10. Other, which ones?

**H10 [Z, max. 1]*.**

**Did you have skin-to-skin contact with your baby after delivery?**

1. Yes,
2. Yes, but it lasted less than 2 hours
3. Not

**H11 [Z, max. 1]*.**

**How would you rate your postpartum lactation experience?**

1. Very good
2. Well
3. Hard to say
4. Badly
5. Very bad

## Part II. Women's motivations and attitudes

**P1 [Z, max. 1]*.**

**Which factors as to your choice of the delivery route would you consider at present?**

|  | Definitely yes | Rather yes | Rather not | Definitely not | I have no opinion |
| --- | --- | --- | --- | --- | --- |
| 1. Willing to minimize pain |  |  |  |  |  |
| 1. Convenience and predictability of planned CC |  |  |  |  |  |
| 1. Extent of cesarean section surgery |  |  |  |  |  |
| 1. Better bond between mother and child |  |  |  |  |  |
| 1. Ensuring better health for your child |  |  |  |  |  |
| 1. Ensuring better health for the mother |  |  |  |  |  |
| 1. Ensuring skin-to-skin contact |  |  |  |  |  |
| 1. Better breastfeeding conditions and opportunities |  |  |  |  |  |
| 1. The impact of the birth route on subsequent pregnancies and deliveries |  |  |  |  |  |
| 1. Faster recovery |  |  |  |  |  |
| 1. Less blood loss |  |  |  |  |  |
| 1. A sense of accomplishment |  |  |  |  |  |
| 1. Strengthening the sense of femininity |  |  |  |  |  |
| 1. Improve ties with your partner |  |  |  |  |  |
| 1. Experiences of previous birth(s) |  |  |  |  |  |
| 1. The experience of previous confinements |  |  |  |  |  |

**P2. [PO, max. 3]*.**

**Which childbirth preparation activities do you find most useful from the perspective of a postpartum person? (Please mark the 3 most important)**

1. Substantive reading on the subject
2. Preparing a birth plan
3. Reading stories of other births after CC
4. Signing up for support groups ( e.g. Facebook, or meetings with other women)
5. Participation in a birthing school
6. Activity during pregnancy
7. Participation in yoga classes
8. Participation in psychotherapy
9. Use of unconventional medicine (e.g., acupressure, herbs)
10. Managing pregnancy with a supportive doctor
11. Childbirth in a hospital where the staff supported my decision
12. Meetings with a supportive midwife
13. Purchase individual midwife care for the duration of childbirth
14. Other (what? ........................)

**P3 [Z, max. 6]*.**

**During your stay in the hospital, who demonstrated particular attitudes toward your decision about the route of delivery? (You may mark more than one answer in each row)**

|  | Enthusiasm | Support | Acceptance | Indifference | Unfriendliness | Criticism | Not applicable |
| --- | --- | --- | --- | --- | --- | --- | --- |
| 1. Midwife in charge of childbirth |  |  |  |  |  |  |  |
| 1. Other midwives |  |  |  |  |  |  |  |
| 1. Physician in charge of childbirth |  |  |  |  |  |  |  |
| 1. Other doctors |  |  |  |  |  |  |  |
| 1. Pediatrician |  |  |  |  |  |  |  |
| 1. Accompanying person |  |  |  |  |  |  |  |

**P4 [PO, max. 12]*.**

**What situations did you encounter during childbirth? (You may mark more than one answer)**

1. Disrespect for my rights
2. Bad attitude of staff towards my decision
3. Rude comments from staff about my decision
4. Persistently urging me to change my decision
5. Not informing me of the situation I am in
6. No induction of labor after CC
7. Lack of mobility during childbirth
8. No epidurals available
9. No possibility of using water immersion
10. No skin-to-skin contact with the newborn
11. I have not encountered such situations
12. Other (what kind?)

**P5 [Z, max. 1]*.**

**How do you evaluate your decision on how to complete the pregnancy?**

1. Very good decision
2. Good decision
3. Hard to say
4. Wrong decision
5. A very bad decision
6. How the pregnancy was completed was not my decision

**P6 [Z, max. 1]***

**If you were pregnant again, what birth route would you choose?**

1. Planned Caesarean section
2. Caesarean section after spontaneous onset of contraction activity
3. Natural childbirth
4. I don't know

**P7 [O]*.**

**Date of delivery (DD-MM-YYYY)**

**P8 [O]*.**

**Which of your expectations for delivery by cesarean section has been realized?**

**P9 [O]*.**

**Which of your expectations for delivery by cesarean section has NOT been realized? Why?**
