# Supplementary material for: The Experience of Women Giving Birth after Cesarean Section—A Longitudinal Observational Study
Source: Healthcare (Basel). 2023 Jun 20;11(12):1806. doi: 10.3390/healthcare11121806 (PMC10297878; doi:10.3390/healthcare11121806)
Supplement: Supplementary file 1 [file healthcare-11-01806-s001.zip › appendixes word/APPENDIX C.docx]

The names and URLs of social media and parenting portals used in this study:

<https://mamadu.pl/>

<https://www.dobrzeurodzeni.pl/start.html>

<https://www.facebook.com/NaturalniePoCesarce>

<https://www.facebook.com/groups/1217598234961330>

<https://www.facebook.com/groups/1538333086443744>

<https://www.facebook.com/groups/1726589684296030>

<https://www.facebook.com/groups/541184229353809>
