# Supplementary material for: The Experience of Women Giving Birth after Cesarean Section—A Longitudinal Observational Study
Source: Healthcare (Basel). 2023 Jun 20;11(12):1806. doi: 10.3390/healthcare11121806 (PMC10297878; doi:10.3390/healthcare11121806)
Supplement: Supplementary file 1 [file healthcare-11-01806-s001.zip › appendixes word/APPENDIX D.docx]

APPENDIX D – Tables and figures

**Table S1.** Characteristics of respondents - sociodemographic data (N=288).

|  | **Emergency CS**  **N=75** | | **VBAC**  **N=122** | | **Elective CS**  **N=64** | | **Lack of consent for TOLAC**  **N=27** | | **p** |  |
| --- | --- | --- | --- | --- | --- | --- | --- | --- | --- | --- |
|  | **n** | **%** | **n** | **%** | **n** | **%** | **n** | **%** |  |  |
| Level of education | | | | | | | | |  |  |
| Lower Secondary | 0 | - | 0 | - | 0 |  | 1 | 3.70 | 0.298 |  |
| Secondary | 8 | 10.96 | 21 | 17.21 | 12 | 18.75 | 6 | 22.22 |  |  |
| Higher | 65 | 89.04 | 101 | 82.79 | 52 | 81.25 | 20 | 74.07 |  |  |
| Relationship | | | | | | | | |  |  |
| Married/Partnership | 73 | 100 | 120 | 98.63 | 62 | 96.88 | 27 | 100 | 0.417 |  |
| Single | 0 | - | 2 | 1.64 | 2 | 3.13 | 0 | - |  |  |
| Age | | | | | | | | | | |
| 21-25 | 4 | 5.48 | 9 | 7.38 | 10 | 15.63 | 3 | 11.11 | 0.080 |  |
| 26-30 | 32 | 43.84 | 54 | 44.26 | 29 | 45.31 | 5 | 18.52 |  |  |
| 31-35 | 30 | 41.10 | 45 | 36.89 | 21 | 32.81 | 14 | 51.85 |  |  |
| 36-40 | 7 | 9.59 | 14 | 11.48 | 4 | 6.25 | 4 | 14.81 |  |  |
| > 40 | 0 | - | 0 | - | 0 | - | 1 | 3.70 |  |  |
| Place of residence | | | | | | | | | | |
| A rural area | 20 | 27.40 | 24 | 19.67 | 13 | 20.31 | 5 | 18.52 |  |  |
| A small city (population less than 50.000) | 11 | 15.07 | 15 | 12.30 | 9 | 14.06 | 2 | 7.14 |  |  |
| A medium city (population 50.000 – 99.999) | 6 | 8.22 | 11 | 9.02 | 9 | 14.06 | 6 | 22.22 |  |  |
| A large city (population 100.000 – 499.999) | 32 | 43.84 | 64 | 52.46 | 28 | 43.75 | 10 | 37.04 | 0.769 |  |
| A very large city (population over 500.000) | 4 | 5.48 | 8 | 6.56 | 5 | 7.81 | 4 | 14.81 |  |  |

CS –cesarean section; VBAC – vaginal birth after cesarean section; TOLAC – trial of labor after cesarean section.

**Table S2.** Mode of delivery vs. Apgar scores and skin-to-skin contact (N=288).

|  | **Emergency CS**  **N=75** | | **VBAC**  **N=122** | | **Elective CS**  **N=64** | | **Lack of consent for TOLAC**  **N=27** | | **χ^2^** | **p** |
| --- | --- | --- | --- | --- | --- | --- | --- | --- | --- | --- |
|  | **n** | **%** | **n** | **%** | **n** | **%** | **n** | **%** |  |  |
| How many Apgar points was the baby rated after birth? | | | | | | | | |  |  |
| 0-3 points | 2 | 2.67 | 1 | 0.82 | 0 | 0.00 | 2 | 7.41 | 15.418 | 0.017 |
| 4-7 points | 6 | 8.00 | 2 | 1.64 | 1 | 1.56 | 0 | 0.00 |  |  |
| 8-10 points | 67 | 89.33 | 119 | 97.54 | 63 | 98.44 | 25 | 92.59 |  |  |
| Did you have skin-to-skin contact with your baby after birth? | | | | | | | | |  |  |
| NO | 41 | 54.67 | 3 | 2.46 | 26 | 40.63 | 9 | 33.33 | 79.335 | <0.001 |
| Yes, but it was less than 2 hours | 13 | 17.33 | 24 | 19.67 | 14 | 21.88 | 6 | 22.22 |  |  |
| YES | 21 | 28.00 | 95 | 77.87 | 24 | 37.50 | 12 | 44.44 |  |  |

CS –cesarean section; VBAC – vaginal birth after cesarean section; TOLAC – trial of labor after cesarean section

**Table S3.** Childbirth experiences about the mode of delivery after cesarean section (N=288).

|  | **Emergency CS**  **N=75** | | **VBAC**  **N=122** | | **Elective CS**  **N=64** | | **Lack of consent for TOLAC**  **N=27** | | **χ^2^** | **p** |
| --- | --- | --- | --- | --- | --- | --- | --- | --- | --- | --- |
|  | **n** | **%** | **n** | **%** | **n** | **%** | **n** | **%** |  |  |
| How do you assess your decision on how to give birth? | | | | | | | | |  |  |
| Bad decision | 0 | 0.00 | 1 | 0. 82 | 0 | 0.00 | 0 | 0.00 | 154.42 | <0.001 |
| Don’t know | 17 | 22.67 | 3 | 2.46 | 16 | 25.00 | 1 | 3.70 |  |  |
| Good decision | 15 | 20.00 | 9 | 7.38 | 17 | 26.56 | 4 | 14.81 |  |  |
| Very good decision | 15 | 20.00 | 108 | 88.52 | 7 | 10.94 | 20 | 74.07 |  |  |
| It wasn't my decision | 28 | 37.33 | 1 | 0.82 | 24 | 37.50 | 2 | 7.41 |  |  |
| How would you rate your lactation experience? | | | | | | | | |  |  |
| Very bad | 0 | 0.00 | 0 | 0.00 | 2 | 3.13 | 2 | 7.41 | 47.415 | <0.001 |
| Bad | 2 | 2.67 | 2 | 1.64 | 9 | 14.06 | 3 | 11.11 |  |  |
| Don’t know | 8 | 10.67 | 4 | 3.28 | 5 | 7.81 | 4 | 14.81 |  |  |
| Good | 20 | 26.67 | 19 | 15.57 | 20 | 31.25 | 6 | 22.22 |  |  |
| Very good | 45 | 60.00 | 97 | 79.51 | 28 | 43.75 | 12 | 44.44 |  |  |
| If you were pregnant again, what mode of delivery would you choose? | | | | | | | | |  |  |
| Elective CS | 8 | 10.67 | 2 | 1.64 | 10 | 15.63 | 16 | 59.26 | 126.62 | <0.001 |
| CS after spontaneous onset of systolic | 14 | 18.67 | 1 | 0.82 | 11 | 17.19 | 6 | 22.22 |  |  |
| Vaginal delivery | 35 | 46.67 | 109 | 89.34 | 27 | 42.19 | 2 | 7.41 |  |  |
| Don’t know | 18 | 24.00 | 010 | 8.20 | 16 | 25.00 | 3 | 11.11 |  |  |

CS –cesarean section; VBAC – vaginal birth after cesarean section; TOLAC – trial of labor after cesarean section.

**Table S4.** Methods of preparing for delivery according to the mode of delivery.

|  | **Emergency CS**  **N=75** | | **VBAC**  **N=122** | | **Elective CS**  **N=64** | | **Lack of consent for TOLAC**  **N=27** | | **χ2** | **p** |
| --- | --- | --- | --- | --- | --- | --- | --- | --- | --- | --- |
|  | **n** | **%** | **n** | **%** | **n** | **%** | **n** | **%** |  |  |
| Substantially -  I read about it | 32 | 42.62 | 52 | 29.69 | 19 | 29.63 | 8 | 42.62 | 4.420 | 0.220 |
| I have prepared a birth plan | 10 | 13.33 | 9 | 7.38 | 3 | 4.69 | 5 | 18.52 | 6.268 | 0.100 |
| I read the stories of other births after CS | 20 | 26.67 | 29 | 23.77 | 11 | 17.19 | 2 | 7.41 | 5.436 | 0.143 |
| I signed up for support groups | 30 | 40.00 | 122 | 43.44 | 18 | 28.13 | 3 | 11.11 | 12.418 | 0.006 |
| I attended childbirth classes | 11 | 14.67 | 14 | 11.48 | 9 | 14.06 | 7 | 25.93 | 3.796 | 0.284 |
| I was physically active during my pregnancy | 75 | 30.67 | 25 | 20.49 | 22 | 34.38 | 9 | 33.33 | 5.368 | 0.147 |
| I attended yoga classes | 3 | 4.00 | 5 | 4.10 | 5 | 0 | 0 | 0 | 3.803 | 0.284 |
| I used psychotherapy | 1 | 1.33 | 4 | 3.28 | 3 | 4.69 | 0 | 0 | 2.328 | 0.507 |
| I used alternative medicine | 1 | 1.33 | 2 | 1.64 | 0 | 0 | 0 | 0 | 1.443 | 0.696 |
| I chose a physician to support my preferences | 20 | 26.67 | 47 | 38.52 | 43 | 67.19 | 21 | 77.78 | 36.607 | <0.001 |
| I chose a hospital to support my preferences | 41 | 54.67 | 82 | 67.21 | 39 | 60.94 | 20 | 74.07 | 4.706 | 0.195 |
| I chose a midwife to support my preferences | 16 | 21.33 | 14 | 11.48 | 14 | 21.88 | 3 | 11.11 | 5.460 | 0.141 |

CS –cesarean section; VBAC – vaginal birth after cesarean section; TOLAC – trial of labor after cesarean section.

**Table S5. Univariate Logistic Regression Analyses for Factors Influencing Mode of Childbirth after cesarean section**

|  | **OR** | **95% CI** | | p |
| --- | --- | --- | --- | --- |
| Minimizing pain | 0.87 | 0.71 | 1.07 | 0.189 |
| Convenience and predictability of ECS | 0.55 | 0.38 | 0.75 | 0.001 |
| The severity of cesarean section | 1.20 | 1.01 | 1.43 | 0.041 |
| A better bond between mother and child | 0.94 | 0.79 | 1.10 | 0.433 |
| Ensuring better health for your child | 1.32 | 1.02 | 1.76 | 0.045 |
| Ensuring better health of the mother | 1.41 | 1.09 | 1.84 | 0.011 |
| Ensuring skin-to-skin contact | 1.14 | 0.91 | 1.44 | 0.265 |
| Better conditions for breastfeeding | 1.15 | 0.95 | 1.41 | 0.164 |
| Influence of the mode of birth on subsequent pregnancies and deliveries | 1.10 | 0.93 | 1.30 | 0.272 |
| Faster recovery | 1.21 | 0.97 | 1.52 | 0.094 |
| Less blood loss | 1.02 | 0.85 | 1.22 | 0.827 |
| A sense of fulfillment | 1.11 | 0.94 | 1.31 | 0.232 |
| Strengthening the sense of femininity | 1.10 | 0.95 | 1.28 | 0.208 |
| Improving your relationship with your partner | 1.07 | 0.89 | 1.30 | 0.452 |
| Previous birth experiences | 1.02 | 0.82 | 1.27 | 0.883 |
| The previous postpartum experience | 0.98 | 0.82 | 1.16 | 0.773 |

aOR – adjusted Odds Ratio, CI – Confidence Interval

Figure S1. Factors of choice of mode of delivery in subsequent pregnancies according to the mode of delivery in the last childbirth.

NOTE: 1. Minimizing pain; 2. Convenience and predictability of ECS; 3. The severity of cesarean section; 4. A better bond between mother and child; 5. Ensuring better health for your child; 6. Ensuring better health of the mother; 7. Ensuring skin-to-skin contact; 8. Better conditions for breastfeeding; 9. Influence of the mode of birth on subsequent pregnancies and deliveries; 10. Faster recovery; 11. Less blood loss; 12. A sense of fulfillment; 13. Strengthening the sense of femininity; 14. Improving your relationship with your partner; 15. Previous birth experiences; 16. The previous postpartum experience.

Figure S2. Factors influencing the choice of mode of delivery- before and after labour

NOTE: 1. Minimizing pain; 2. Convenience and predictability of ECS; 3. The severity of cesarean section; 4. A better bond between mother and child; 5. Ensuring better health for your child; 6. Ensuring better health of the mother; 7. Ensuring skin-to-skin contact; 8. Better conditions for breastfeeding; 9. Influence of the mode of birth on subsequent pregnancies and deliveries; 10. Faster recovery; 11. Less blood loss; 12. A sense of fulfillment; 13. Strengthening the sense of femininity; 14. Improving your relationship with your partner; 15. Previous birth experiences; 16. The previous postpartum experience.
